# Label-Free Quantitative Proteomics Combined with Biological Validation Reveals Activation of Wnt/β-Catenin Pathway Contributing to Trastuzumab Resistance in Gastric Cancer

**DOI:** 10.3390/ijms19071981

**Published:** 2018-07-06

**Authors:** Wenhu Liu, Jiangbei Yuan, Zhenzhong Liu, Jianwu Zhang, Jinxia Chang

**Affiliations:** 1 Department of Medicinal Chemistry, Department of Pharmacology, School of Pharmacy, North Sichuan Medical College, Nanchong 637100, China; wenhuliu@cqu.edu.cn (W.L); jianwuzhang@nsmc.edu.cn (J.Z); 2 Innovative Platform of Basic Medical Sciences, Department of Microbiology and Immunology, School of Basic Medical Sciences, North Sichuan Medical College, Nanchong 637100, China; 3 School of Pharmaceutical Sciences and Innovative Drug Research Center, Chongqing University, Chongqing 401331, China; yuanjiangbei@cqu.edu.cn; 4 Department of Preventive Medicine, North Sichuan Medical College, Nanchong 637100, China; liuzhenzhong@nsmc.edu.cn

**Keywords:** gastric cancer, trastuzumab resistance, Wnt/β-catenin pathway, EMT, label-free quantitative proteomics

## Abstract

Resistance to trastuzumab, which specifically target HER2-positive breast and gastric cancer, can develop ultimately in cancer patients. However, the underlying mechanisms of resistance in gastric cancer have not been fully elucidated. Here, we established trastuzumab-resistant MKN45 and NCI N87 gastric cancer sublines from their parental cells. The resistant cells exhibited characteristics of epithelial-mesenchymal transition (EMT) and acquired higher migratory and invasive capacities. To exploit the activated pathways and develop new strategies to overcome trastuzumab resistance, we investigated MKN45 and MKN45/R cells via label-free quantitative proteomics, and found pathways that were altered significantly in MKN45/R cells, with the Wnt/β-catenin pathway being the most significant. We further confirmed the activation of this pathway by detecting its key molecules in MKN45/R and NCI N87/R cells via Western blot, in which Wnt3A, FZD6, and CTNNB1 increased, whereas GSK-3β decreased, manifesting the activation of the Wnt/β-catenin pathway. Correspondingly, inhibition of Wnt/β-catenin pathway by ICG-001, a specific Wnt/β-catenin inhibitor, preferentially reduced proliferation and invasion of trastuzumab-resistant cells and reversed EMT. Concurringly, CTNNB1 knockdown in stable cell lines potently sensitized cells to trastuzumab and induced more apoptosis. Taken together, our study demonstrates that the Wnt/β-catenin pathway mediates trastuzumab resistance, and the combination of Wnt/β-catenin inhibitors with trastuzumab may be an effective treatment option.

## 1. Introduction

Gastric carcinoma, which is the fourth leading cause of cancer death worldwide, has half of its incidences detected in East Asian countries, with mortality rates being higher than other countries [1,2]. The search for the adequate and novel treatment has been a constant quest in this regard. Human epidermal growth factor receptor-2 (HER-2), a transmembrane tyrosine kinase receptor belonging to the family of epidermal growth factor receptor (EGFR), is an important gastric cancer target encoded by the *ErbB2* gene located on chromosome 17q21 [3,4]. A positive correlation exists, as inferred from numerous studies, between HER-2 over-expression and cancer cell proliferation, malignancy, metastasis, and poor outcomes [5,6,7]. HER-2 over-expression and/or *ErbB2* gene amplification (20% of gastric cancer cases) represents a negative predictor of response to chemotherapy and a positive factor to anti-HER2 agents [4]. Previous studies have confirmed that HER-2 activation can be perceived as a trigger of multiple cell signal transduction pathways, which promotes aberrant cell proliferation and drug resistance [8,9].

As a result of rapid advancement in the field of tumor biology, attention has been focused on the new modality of molecular targeted therapy for advanced cancer [10,11]. Molecular-targeted drugs such as trastuzumab (Herceptin^®^), a humanized monoclonal antibody interfering with the extracellular domain of HER2/neu receptor, has been proved to be beneficial in patients with HER2-positive advanced gastric and breast cancer in clinical treatment [12,13]. Unfortunately, the acquired resistance could hinder the effectiveness of trastuzumab [14,15].

In clinical practice, acquired resistance can be a major barrier for antineoplastic agents. Some potential mechanisms of trastuzumab resistance include mutational activation of the phosphatidylinositide 3-kinase (PI3K)/AKT pathway [16], up-regulation of insulin-like growth factor receptor (IGFR) and hetero-dimerization of IGFR/HER-2 [17,18], loss of phosphatase and tensin homolog gene (PTEN) function [19], and accumulation of truncated HER-2 receptor (p95HER-2) [20], all of which have been verified as principal pathways in breast cancer. Although gastric cancer does possess some of these pathway modulations, there are some gastric cancer-specific mechanisms too. For instance, over-expression of miR-223 in miR-223/FBXW7 pathway [21], up-regulation of fibroblast growth factor receptor 3 (FGFR3)/AKT axis [22], activation of β2-adrenergic receptor (β2-AR) signaling, and loss of HER-2 [23,24] are some of the mechanisms. As opposed to breast cancer, gastric cancer still lacks extensive research in signaling pathways which mediate acquired trastuzumab resistance.

Mass spectrometry-based proteomics has emerged as a powerful tool for large-scale protein analysis in biological research [25,26]. Ding et al. have developed a novel technique in recent years named label-free quantification workflow (Fast-quan) for protein quantification, in which 7000 proteins can be detected and quantified within 12 h of mass spectrometry running time [27]. Here, the trastuzumab-resistant sublines, MKN45/R and NCI N87/R, were obtained by continuous exposure to increasing doses of trastuzumab up to 80 μg/mL. We proved that there is an association between acquirement of trastuzumab resistance and EMT. We also performed label-free proteome profiling of MKN45 and MKN45/R, analyzed differential proteins and explored the corresponding pathways using bioinformatics techniques. In addition, a series of biological validation were conducted and the activation of canonical Wnt/β-catenin pathway in both MKN45/R and NCI N87/R cells was confirmed. Suppression of Wnt/β-catenin signaling by ICG-001 decreased viability and induced apoptosis of trastuzumab resistant cells in a dose-dependent manner and reversed EMT. Also, knockdown of β-catenin suppressed cell proliferation and enhanced sensitivity to trastuzumab of resistant cells, implying this pathway to be a possible treatment target for trastuzumab-resistant gastric carcinoma.

## 2. Results

### 2.1. Establishment of Trastuzumab-Resistant Gastric Cancer Cell Lines

We employed Western blot to detect the expression of HER-2 in all six gastric cancer cell lines, including NCI N87, MKN45, MKN28, BGC823, MGC803, and SGC7901, with a relatively high level being observed in MKN45 and NCI N87 cells (Figure S1a). To simulate the in vivo mode of resistance, we treated MKN 45 and NCI N87 cell lines with increasing doses of trastuzumab for five months. Once the drug concentration level reached up to 80 μg/mL, trastuzumab-resistant sublines MKN45/R and NCI N87/R were then harvested. The IC_50_ values of MKN45 and MKN45/R cells were 56.48 and 414.52 μg/mL, and that of NCI N87 and NCI N87/R cells were 73.22 and 436.17 μg/mL, respectively (Figure S1b,c). The resistance index of MKN45/R and NCI N87/R cell lines for trastuzumab were 7.34 and 5.96 respectively, indicating the remarkable resistance of MKN45/R and NCI N87/R cells to trastuzumab in vitro. Furthermore, we detected cleaved poly ADP-ribose polymerase (PARP) levels in parental and trastuzumab-resistant cells after trastuzumab treatment (0, 60, 80 μg/mL) by Western blot; consistent with the inhibition rate of trastuzumab on cell viability, it showed an increase in a dose-dependent manner, with a more prominent increase observed in parental cells (Figure S1d).

To determine if the acquired resistance of the above mentioned sublines was related to HER-2 over-expression, HER-2 level in parental cells and resistant sublines were detected by Western blot which showed no statistical difference. This implied different mechanisms of acquired resistance other than HER-2 expression change [28] (Figure S1e).

### 2.2. Trastuzumab-Resistant Gastric Cancer Cells Exhibit an EMT-Like Phenotypic Change

**Figure 1:**
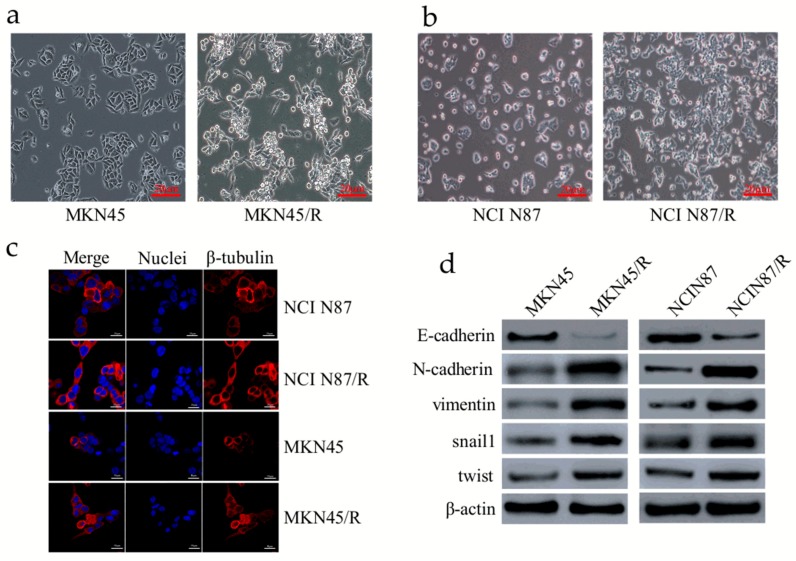

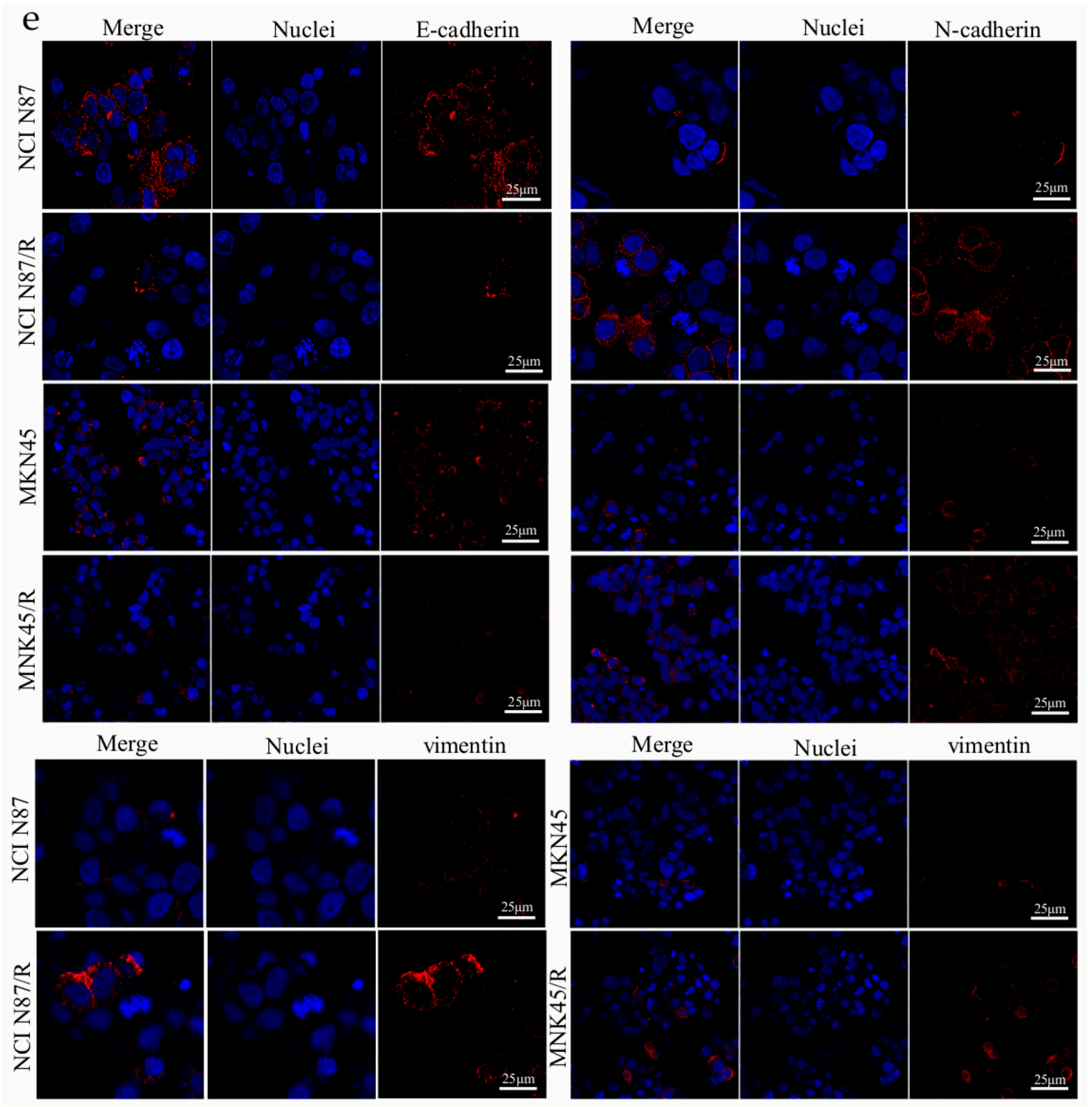
Trastuzumab resistance is associated with epithelial-mesenchymal transition (EMT) in gastric cancer cells. (**a**,**b**) MKN45/R and NCI N87/R cells showed morphological changes when compared with their parental cells respectively; (**c**) Images of cells stained with antibodies against β-tubulin (red), and nuclei stained with DAPI (blue) (original magnification ×10 for NCI N87 and NCI N87/R cells, ×20 for MKN45 and MKN45/R cells respectively); (**d**) Expression of E-cadherin, N-cadherin, vimentin, snail1, and twist in trastuzumab-resistant cells and their parental cells as detected by western blot; (**e**) NCI N87, NCI N87/R, MKN45, and MKN45/R cells were labeled with antibodies against E-cadherin, N-cadherin, and vimentin. Binding was detected by Alexa Fluor^®^ 594-labeled secondary antibody. Staining of nuclei was done with 1 μg/mL DAPI.

EMT is of vital importance in many cancer-related biological events, which contributes to invasion, metastasis, and drug resistance of cancer [29]. When compared with parental cells, MKN45/R and NCI N87/R cells exhibited an EMT-like phenotype (Figure 1a,b). Immunofluorescence staining of β-tubulin also revealed the morphological change of NCI N87/R and MNK45/R cells (Figure 1c). Results of Western blot and immunofluorescence revealed that expression of epithelial marker E-cadherin was dramatically down-regulated while mesenchymal markers N-cadherin and vimentin were markedly up-regulated in MKN45/R and NCI N87/R cells when compared with their parental cells (Figure 1d,e). Moreover, EMT-related transcription factors, including snail1 and twist, were significantly up-regulated in trastuzumab-resistant cells (Figure 1d). These results suggest an EMT phenotypic conversion in resistant cells.

### 2.3. Trastuzumab Resistant Cells Exhibit Increased Capacity of Migration and Proliferation

**Figure 2:**
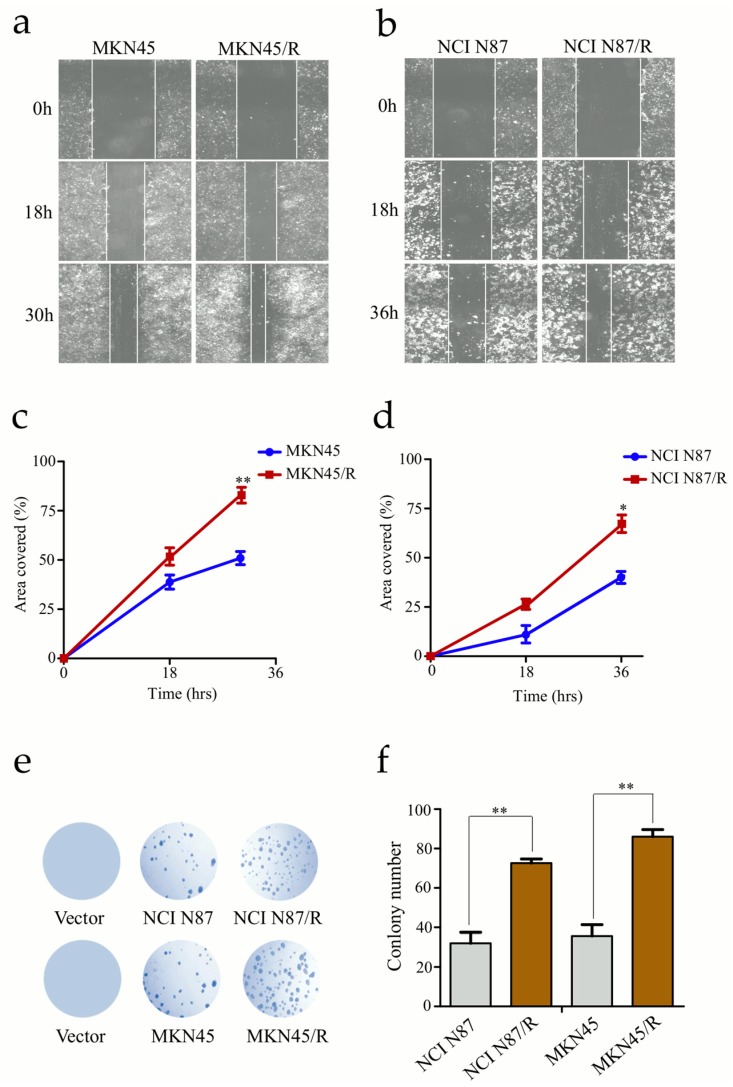
Trastuzumab resistant cells exhibit aggressive migration. (**a**,**b**) The confluent cell monolayers were wounded by scraping and the closure of the wounded areas was monitored. The images of the wounds were taken at different time points; (**c**,**d**) Migration activities were analyzed by measuring the distance cells traveled toward the center of the wound; (**e**,**f**) Colony formation assays were performed and colony numbers were counted and analyzed. All experiments were performed in triplicate. * *p* < 0.05, ** *p* < 0.01 vs. controls.

Concomitant enhancement in cellular migration and proliferation activities is commonly along with acquisition of resistance by cancer cells. We evaluated migration abilities of the resistant cells by wound healing assays. In contrast to parental cells, resistant cells showed prominently higher migration potential evidenced by the rapid and complete wound healing (Figure 2a–d). Moreover, by means of plate colony formation assay we detected proliferative potency of trastuzumab resistant cells which formed bigger and multiple colonies than that of parental cells (Figure 2e,f). These data indicated that trastuzumab-resistant gastric cancer cells acquired higher malignant behaviors than their parental cells.

### 2.4. Label-Free Proteomic Profiling of MKN45 and MKN45/R Cells

To explore signal pathways that mediate trastuzumab resistance in trastuzumab-resistant cells, we implemented label-free quantitative proteomics profiling for a proteome comparison between MKN45 and MKN45/R cells as according to our previous protocols [30]. Three biological repeats of these two cell lines were processed respectively. Protein abundance was quantified using intensity based absolute quantification (iBAQ) and normalized by the fraction of total (FOT) which represented standardized value of a protein across samples. The FOT was multiplied by 10^5^ for the ease of presentation. We detected 7645 proteins with at least two unique peptides at 1% peptide FDR. From this pool, 5996 proteins were identified at 1% protein FDR (Figure S2, Table S1) with 5461 proteins being detected from three out of six experiments which were subsequently selected for bioinformatics analyses (Figure S2, Table S2). The high correlation (*r* ≥ 0.85) of each pair of the three repeats displayed a good repeatability (Figure S3). A principal component analysis (PCA) revealed that samples of MKN45 and MKN45/R were completely separated, and three biological repeats of each cell line were well-clustered (Figure S4). The distribution of these proteins in different ratio range are shown in Figure S5.

### 2.5. Identification of Differentially Expressed Proteins and Enrichment Analysis

**Figure 3:**
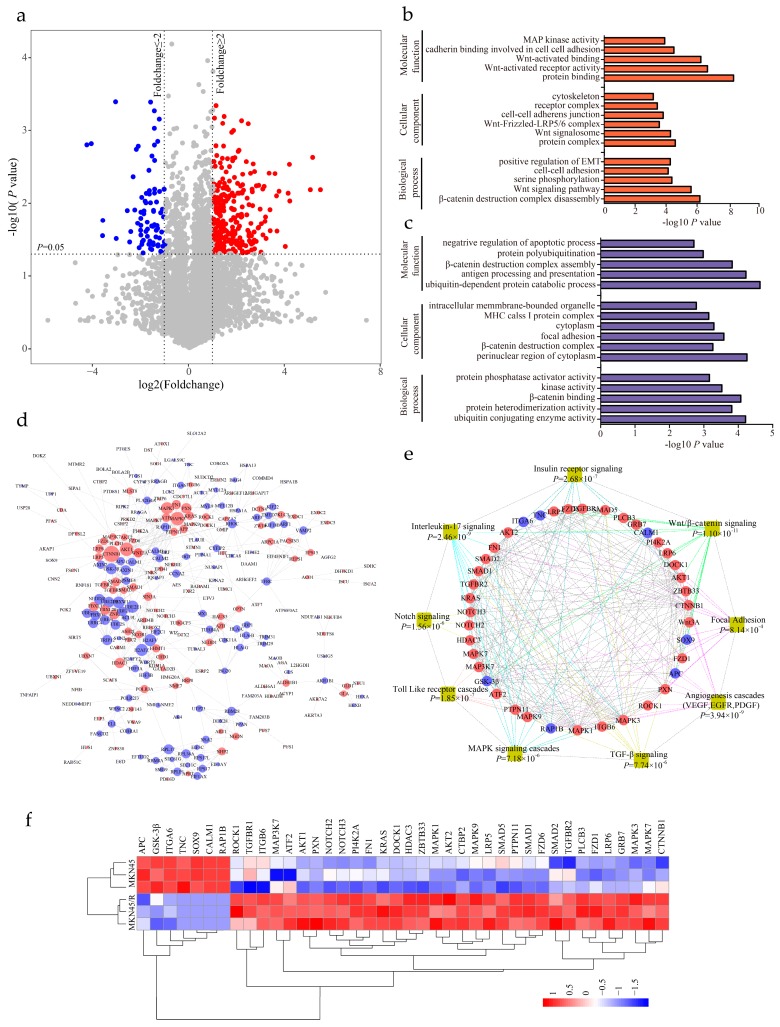
Bioinformatics analysis of differentially expressed proteins and significantly altered pathways. (**a**) Differentially expressed proteins in MKN45 and MKN45/R cells are illustrated by volcano plot. The mean ratios of three biological repeats (MKN45/R group/MKN45 group) are plotted in log2 scale (*x*-axis) against the corresponding −log10 *p* value (*y*-axis). The vertical dotted lines mark twofold change and horizontal dotted lines represent cutoff *p* value = 0.05. Proteins which showed fold changes greater than 2 or less than 0.5 and *p* < 0.05 are considered as up- or down-regulated and marked in red and blue, respectively. The gray dots are considered as no significant change; (**b**,**c**) Gene ontology (GO) enrichment analysis of upregulated (red bar) and downregulated proteins (blue bar) involved in cell component, molecular function and biological process; (**d**) Network analysis of differentially expressed proteins performed by Cytoscape. Red and blue nodes represent increased and decreased proteins respectively. Sizes of nodes correspond to numbers of interacting neighbors; (**e**) WebGestalt analysis identified major biological pathways in which differential proteins are involved. Each colored line indicates a different pathway; (**f**) Heat map visualization of forty differential proteins identified from several pathways in both MKN45 and MKN45/R cells. The increased and decreased proteins are represented by range of red and blue intensities, respectively.

We performed paired two-tailed student’s *t* tests (the dataset showed a normal distribution (Figure S5) to identify differentially expressed proteins with statistical significance. A volcano plot elucidated differential protein abundance against the corresponding *p* value obtained from *t* test (Figure 3a, Table S3). Compared with MKN45, the quantitative values of 286 proteins (5.24% of the proteome) displayed a more than twofold increase in MKN45/R cells (*p* value < 0.05, and marked as red dots), whereas 211 proteins (3.86% of the proteome) showed more than twofold decrease in MKN45/R cells (*p* value < 0.05, and marked as blue dots). The rest of the 4964 proteins (90.90% of the proteome) were considered as no significant change. The increased and decreased proteins were subjected to gene ontology (GO) terms enrichment analysis by the WebGestalt (http://www.webgestalt.org). The enrichment analysis of up- or down-regulated proteins in cellular component, biological process and molecular function were shown in Figure 3b,c respectively. Notably, up-regulated proteins in MKN45/R cells were annotated as residing in protein complex, Wnt signalosome, Wnt-Frizzled-LRP5/6 complex and cell-cell adherens junction, whereas down-regulated proteins in MKN45/R cells were annotated as perinuclear region of cytoplasm, β-catenin destruction complex, and focal adhesion. Regarding biological processes, up-regulated proteins include those involved in regulation of β-catenin destruction complex disassembly, Wnt signaling pathway, serine phosphorylation, and positive regulation of EMT, while down-regulated proteins were mainly shown as ubiquitin conjugating enzyme activity, protein hetero-dimerization activity and β-catenin binding. With regard to molecular functions, upregulated proteins are enriched in protein binding, Wnt-activated receptor activity, Wnt-activated binding, and cadherin binding involved in cell-cell adhesion, whereas down-regulated proteins function in ubiquitin-dependent protein catabolic process, antigen processing and presentation, and β-catenin destruction complex assembly, etc. the STRING database (https://string-db.org/) was then utilized to uncover the relationship between the increased and decreased proteins. Interactions between them with high confidence (scores > 0.7) were shown using Cytoscape (v.3.6.0). Red and blue nodes represented up- and down-regulated proteins respectively, nodes with more than 10 interacting neighbors were displayed in large size while nodes with 5–10 neighbors were displayed in medium size. We noticed that CTNNB1, AKT1, Wnt3A, and MAPK represent main hubs formed by up-regulated proteins, and GSK-3β, Axin1/2, and UBE family proteins represented primary hubs formed by down-regulated ones (Figure 3d). Enrichment analysis displayed that some signaling pathways showed remarkable changes in MKN45/R cells, which are correlated with cancer metastasis, invasion and drug resistance, with main interaction nodes formed by components of Wnt/β-catenin (*p* = 1.10 × 10^−11^), insulin receptor (*p* = 2.68 × 10^−7^), interleukin-17 (*p* = 2.46 × 10^−9^), Notch (*p* = 1.56 × 10^−6^), Toll like receptor cascades (*p* = 1.85 × 10^−7^), MAPK cascades (*p* = 7.18 × 10^−6^), TGF-β (*p* = 7.74 × 10^−6^), angiogenesis cascades (*p* = 3.94 × 10^−9^), and focal adhesion (*p* = 8.14 × 10^−4^) signaling pathways (Figure 3e). Quantification of these proteins are represented in heat map (Figure 3f).

### 2.6. Label-Free Quantitative Proteomics Profiling Reveals the Activation of Canonical Wnt/β-Catenin Signaling Pathway in Trastuzumab-Resistant Cells

**Figure 4:**
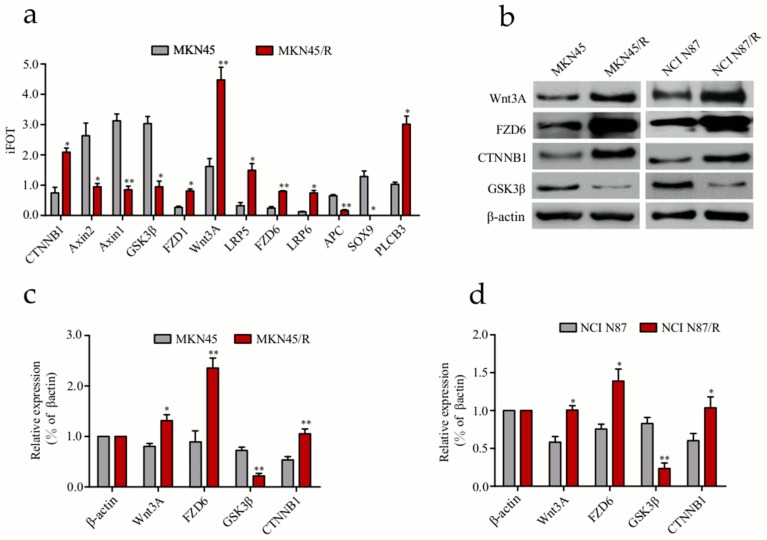
Validation of differentially expressed proteins in Wnt/β-catenin signaling pathway. (**a**) Twelve Wnt/β-catenin pathway components differentially expressed in MKN45/R and MKN45 cells were measured by mass spectrometry; (**b**) Expression of some Wnt/β-catenin pathway proteins detected by western blot. β-actin was used as a loading control; (**c**,**d**) Quantification of Western blot signals in two groups of cell lines. Three independent biological replicates were shown as mean ± SEM. * *p* < 0.05, ** *p* < 0.01 vs. control.

Our results showed that some signaling components exhibited prominent changes in MKN45/R cells while changes in Wnt/β-catenin pathway molecules attracted our attention. Mass spectrometry identified twelve key signaling molecules associated with Wnt/β-catenin pathway (Table S4), including CTNNB1, Axin1/2, GSK-3β, FZD1/6, Wnt3A, LRP5/6, APC, SOX9, and PLCB3 which displayed prominent changes in protein quantification (Figure 4a). Next, we confirmed the expression alteration of Wnt3A, FZD6, CTNNB1, and GSK-3β in MKN45/R and NCI N87/R cells via Western blot (Figure 4b). Consistent with our mass spectrometry data, an increase of Wnt3A, FZD6 and CTNNB1, and a decrease of GSK-3β was observed in these two resistant cell lines as compared to their parental cells (Figure 4c,d). In addition, WebGestalt revealed the connection of Wnt/β-catenin signaling molecules to multiple diseases or pathophysiology of diseases, out of which the foremost nine include adenomatous polyposis coli, hepatoblastoma, neoplasm invasiveness, fibroma, osteoarthritis, colonic neoplasms, tumor angiogenesis, neoplasm metastasis, and pancreatic neoplasms (Figure S6a). Following this, we explored the relationship between up- and down- regulated proteins with the STRING database to display protein-protein interaction network. CTNNB1 represents a primary center in the network involving differential proteins in Wnt/β-catenin pathway, and nine of them, FZD1/6, LRP5/6, Wnt3A, GSK-3β, APC and Axin1/2, constitute the center of the network with high interaction confidence scores (above > 0.9) (Figure S6b). These signaling molecules were demonstrated in an interactive network of canonical Wnt/β-catenin pathway (Figure S6c).

### 2.7. ICG-001 Reduces Viability, Induces Apoptosis and Inhibits β-Catenin Phosphorylation of MKN45/R and NCI N87/R Cells

**Figure 5:**
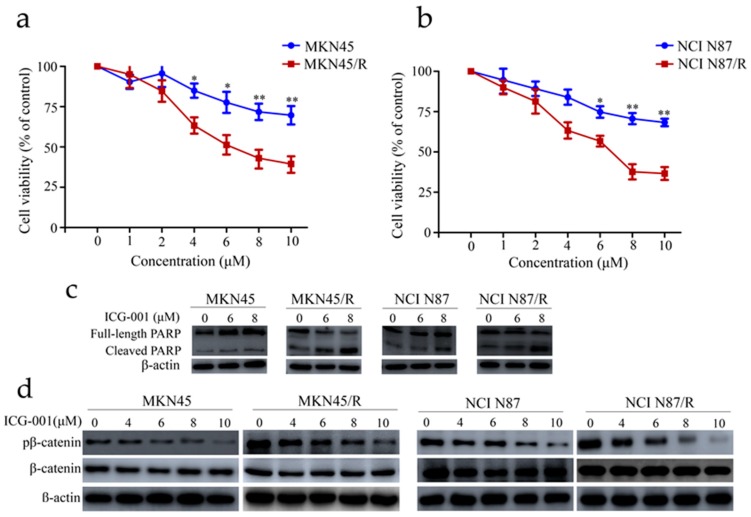

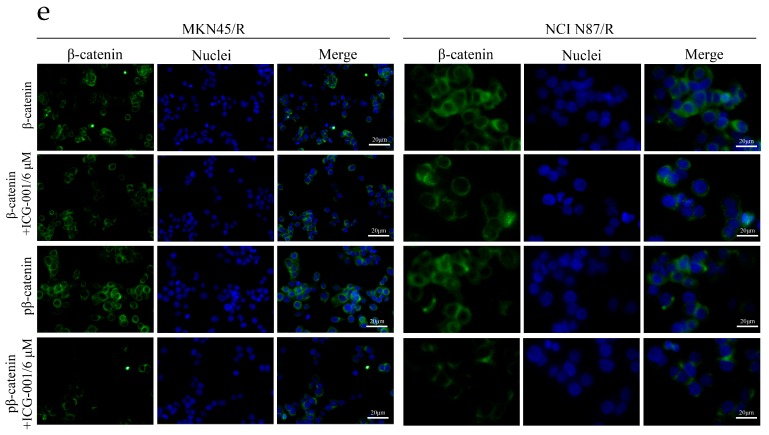
ICG-001 preferentially inhibited cell viability and phosphorylation of β-catenin and induced apoptosis in trastuzumab resistant cells. (**a**,**b**) Cells cultured for 24 h were either treated with or without ICG-001 at the indicated concentrations or (dimethyl sulfoxide) DMSO, which was set as control. Cell viability was determined by CCK-8 assay 72 h later. Results expressed as % control represented the mean of three experiments. * *p* < 0.05, ** *p* < 0.01 vs. controls; (**c**) Cells exposed to ICG-001 for 24 h under different concentrations of (0, 6, 8 μM) were analyzed by Western blot for full-length PARP and cleaved PARP, with β-actin being a loading control; (**d**) Cells were exposed to ICG-001 for 24 h at the indicated concentrations and with DMSO as control, and cell lysates were probed with phosphor- and total antibodies of β-catenin signaling pathway. β-actin was used as a loading control. The representative images were cropped and shown; (**e**) MKN45/R and NCI N87/R cells were treated with ICG-001 (6 μM) for 24 h and DMSO as control which was followed by collection for immunofluorescence. Nuclear staining was performed using DAPI (blue), and total β-catenin and phosphor β-catenin were stained by Alexa Fluor^®^ 488-labeled secondary antibody (green). Slides were imaged using ×20 lens of Leica DMi8 fluorescent microscope.

ICG-001 is known to be a novel selective inhibitor of the canonical Wnt/β-catenin pathway which targets various cancer cell lines [31,32]. It disrupts the interaction of β-catenin with T-cell factor/lymphoid enhancer factor (TCF/LEF) by binding to the transcriptional coactivator cyclic adenosine monophosphate (AMP) response element-binding protein (CBP) and antagonizes Wnt/β-catenin/TCF-mediated transcription. To examine the inhibitory effect of ICG-001 on Wnt/β-catenin pathway in gastric cancer cells, MKN45, MKN45/R, NCI N87, and NCI N87/R cells were cultured under different concentrations of ICG-001 for 72 h. ICG-001 suppressed MKN45, MKN45/R, NCI N87, and NCI N87/R cell proliferation in a dose-dependent manner, however, the effect was more prominent in MKN45/R and NCI N87/R cells at all dosages (Figure 5a,b). It reached statistical significance at 4 μM with cell viabilities of 83.4% and 64.7% in MKN45 and MKN45/R, and 6 μM with cell viabilities of 81.6% and 66.3% in NCI N87 and NCI N87/R, respectively (*p* < 0.05) (Figure 5a,b). In keeping with the inhibition of ICG-001 on cell viability, western blot also showed a dose-dependent increase of cleaved PARP after ICG-001 treatment, with a more prominent increase observed in resistant sublines (Figure 5c). These results indicated that trastuzumab-resistant gastric cancer cells became more sensitive to β-catenin inhibition by ICG-001 than their parental cells, which coheres with its dependence on the β-catenin pathway for survival. To further confirm the activation of Wnt/β-catenin pathway which mediates trastuzumab resistance, Western blot was conducted to detect the phosphorylation of β-catenin, which is a known signaling event in the canonical Wnt/β-catenin pathway. As anticipated, phosphor β-catenin were higher in MKN45/R and NCI N87/R cells than their parental cells, and the decrease of phosphor β-catenin in MKN45/R after ICG-001 treatment was more pronounced between 6 μM and 10 μM (Figure 5d). Similar results were observed between NCI N87 and NCI N87/R cells (Figure 5d). Moreover, immunofluorescence assay also confirmed the significant decrease of phosphor β-catenin in MKN45/R and NCI N87/R cells following treatment with ICG-001 (Figure 5e). To summarize, our results revealed the activation of canonical Wnt/β-catenin pathway in MKN45/R and NCI N87/R cells which can be preferentially inhibited by ICG-001.

### 2.8. ICG-001 Inhibits the Invasion of Trastuzumab-Resistant Cells

**Figure 6:**
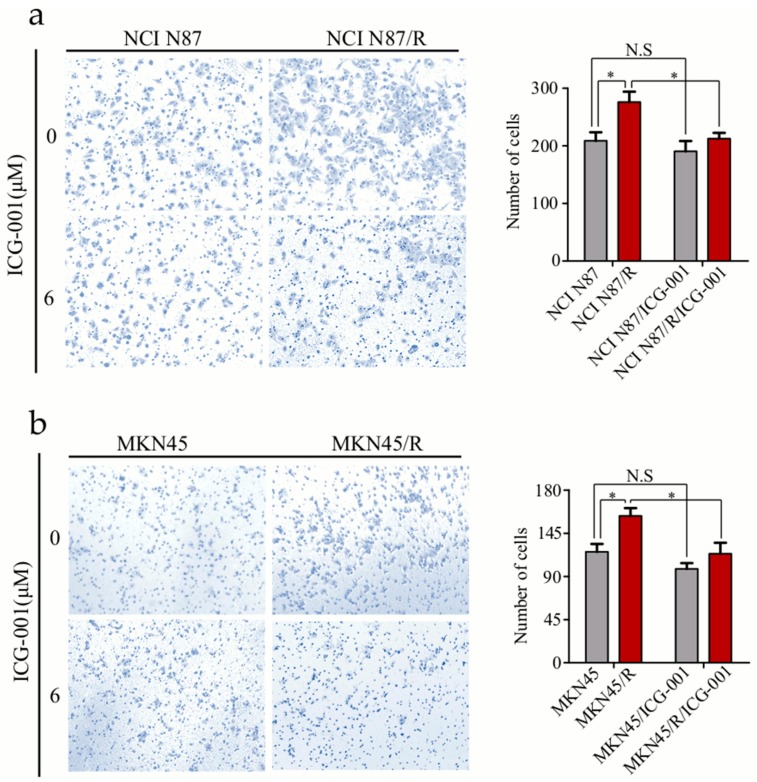
ICG-001 inhibits invasion of trastuzumab resistant cells. Cells were cultured with or without ICG-001 under indicated concentrations, and their invasive ability was evaluated by transwell assay. (**a**,**b**) (Left panels) Representative image of cell invasion (original magnification 100×). (**a**,**b**) (Right panels) Quantitative results of invasion assays, the results are expressed as mean ±SEM of three independent experiments. * *p* < 0.05 vs. controls. N.S = no statistical significance.

Cancer cells usually become more invasive after the acquisition of drug resistance. Here we evaluated how ICG-001 inhibited invasion of MNK45/R and NCI N87/R cells by transwell assays. Without ICG-001, more MKN45/R and NCI N87/R cells passed through the polycarbonate membrane than their parental cells (*p* < 0.05). However, with ICG-001 treatment, invasion of MKN45/R and NCI N87/R cells was remarkably inhibited (*p* < 0.05) while invasion of MKN45 and NCI N87 cells was not (*p* > 0.05) (Figure 6a,b). These results indicated that activation of Wnt/β-catenin pathway facilitates invasion of MKN45/R and NCI N87/R cells in vitro, and ICG-001 can preferentially inhibit the invasion of MKN45/R and NCI N87/R cells.

### 2.9. Knockdown of β-Catenin in Trastuzumab-Resistant Cells Suppresses Proliferation and Enhances Trastuzumab Sensitivity

**Figure 7:**
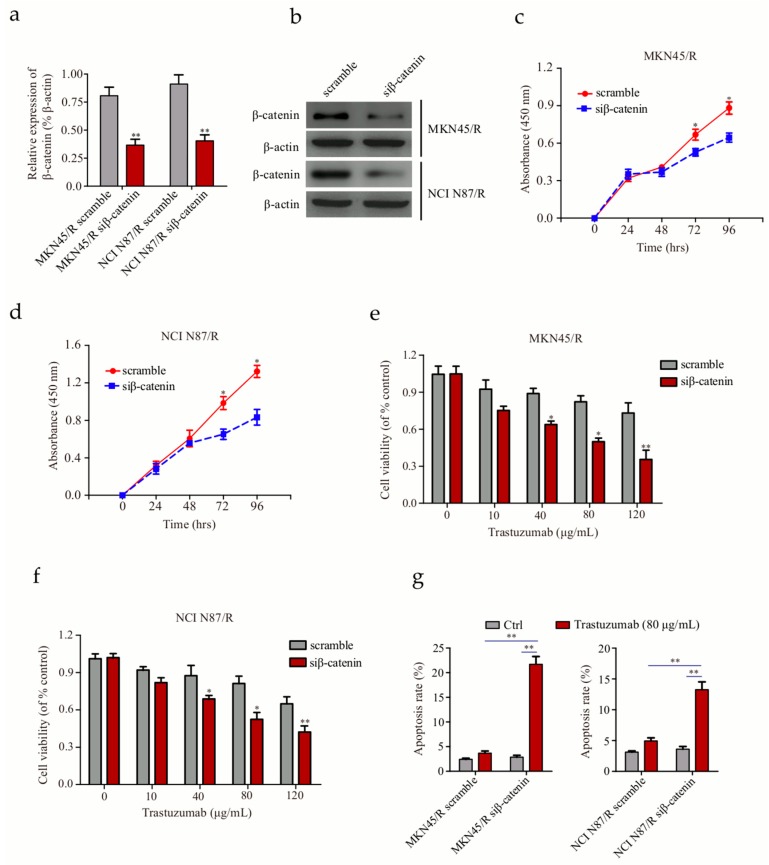
Knockdown of β-catenin enhances the sensitivity of trastuzumab-resistant gastric cancer cells to trastuzumab (**a**,**b**) Evaluation of β-catenin knockdown in trastuzumab-resistant MKN45 and NCI N87 cells by real time-polymerase chain reaction (RT-PCR) and Western blot respectively; (**c**,**d**) Knockdown of β-catenin via small interfering RNA (siRNA) targeting β-catenin (siβ-catenin) decreased proliferation of MKN45/R and NCI N87/R cells; (**e**,**f**) Knockdown of β-catenin induced a decrease of living cells after trastuzumab treatment; (**g**) Detection of cell apoptosis by Annexin V/PI staining upon treatment with 80 μg/mL trastuzumab for 96 h. All data represent three independent experiments. * *p* < 0.05, ** *p* < 0.01 vs. control.

To ascertain whether the Wnt/β-catenin pathway is critical for maintainence of trastuzumab resistance, we established stable cell lines via lentiviral small interfering RNA (siRNA) mediated β-catenin knockdown. The efficiency of β-catenin knockdown was assessed by real time-polymerase chain reaction (RT-PCR) and Western blot respectively (Figure 7a,b). We compared the proliferation activity of cells transfected with scramble control and siRNA targeting β-catenin. As expected, knockdown of β-catenin in trastuzumab-resistant MKN45/R and NCI N87/R cells led to a decrease in proliferation (Figure 7c,d); correspondingly, cell viability decreased after treatment with trastuzumab (Figure 7e,f), implicating the direct correlation between Wnt/β-catenin pathway and trastuzumab resistance. Moreover, Annexin V-FITC/PI staining showed 5.9- and 2.8-fold increases in cell apoptosis upon trastuzumab treatment in MKN45/R siβ-catenin and NCI N87/R siβ-catenin cells compared to their controls respectively (Figure 7g). These data suggest that knockdown of β-catenin causes a decrease in cell proliferation, an increase in trastuzumab sensitivity and enhancement in apoptosis of gastric cancer cells.

### 2.10. Inhibition of Wnt/β-Catenin Signaling by ICG-001 Reverses EMT in Trastuzumab Resistant Cells

**Figure 8:**
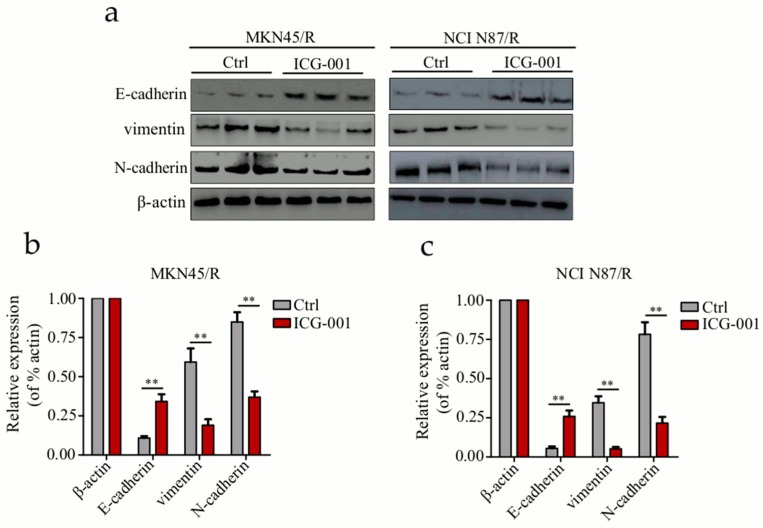
Inhibition of Wnt/β-catenin pathway by ICG-001 reverses EMT in trastuzumab resistant gastric cancer cells. (**a**) Western blot analysis of E-cadherin, N-cadherin, and vimentin in trastuzumab resistant cells before and after 48 h ICG-001(6 μΜ) treatment; (**b**,**c**) Expression of E-cadherin, N-cadherin and vimentin were normalized against β-actin. The results are expressed as mean ± SEM of three independent experiments. ** *p* < 0.01 vs. control.

To gain further insights into the inhibition of Wnt/β-catenin pathway in regulating the EMT process, we carried out Western blot to examine the expression of E-cadherin, N-cadherin, and vimentin which are EMT-related markers. Comparing with the control group, E-cadherin was significantly up-regulated, while N-cadherin and vimentin were down-regulated in MKN45/R and NCI N87/R cells after treatment with ICG-001 for 48 h (Figure 8a–c). Our results indicated that ICG-001 reversed the decrease of E-cadherin, and the increase of N-cadherin and vimentin in MKN45/R and NCI N87/R cells, manifesting that inhibition of β-catenin signaling by ICG-001 reverses EMT in trastuzumab-resistant gastric cancer cells.

## 3. Discussion

Tratuzumab, which has been used in clinical therapy for 20 years, is one of the most effective anti-HER2 antibodies in breast and gastric cancer, yet it shows a limited curative effect due to tumor acquired resistance. Although multiple mechanisms of trastuzumab resistance have been proposed in breast cancer, it is ambiguous whether similar mechanisms exist in gastric cancer. Therefore, understanding the molecular mechanisms and identifying the phenotype of trastuzumab resistance in gastric cancer is of great significance in developing novel therapeutic strategies. Targeted DNA exome and messenger RNA (mRNA) sequencing has been extensively applied in analyses and identification of the genome and transcriptome; however genomic, transcriptomic, and proteomic data was shown to have a poor coherence [33,34]. Without doubt, measurement of protein is more efficient than detecting DNA and mRNA for mining actionable drug targets. Owing to recent progress in liquid chromatography coupled to high resolution mass spectrometry instrumentation and techniques, proteome profiling has become a fairly precise method. Advanced mass spectrometry-based proteomics in our previous research demonstrated that activated mTOR signaling contributes to trastuzumab resistance by inducing NCI N87 cells at low dosage (10 μg/mL) of trastuzumab. In the present study, we acquired a pair of trastuzumab resistant gastric cancer cell lines (MKN45/R and NCI N87/R) by using a higher concentration of trastuzumab (80 μg/mL). Label-free proteome profiling was employed to identify specific signaling pathways that mediated trastuzumab resistance using MKN45 and MKN45/R cell lines. Intriguingly, the Wnt/β-catenin pathway rather than mTOR signaling is the most dominant activated signaling pathway that mediates trastuzumab resistance. Since MKN45 and NCI N87 were derived from different types of gastric cancer that possess variant genetic backgrounds, it is possible that they may acquire resistance via different mechanisms. Besides, the activation of different pathways may implicate a shift of survival signaling in trastuzumab-resistant gastric cancer cells at different dosages of trastuzumab.

Wnt/β-catenin pathway is known to play an important role in organogenesis and pathogenesis of a variety of diseases which includes cancer as well [35,36]. Roles of canonical Wnt/β-catenin pathway in regulating tumor cell properties, such as self-renewal, motility and tumor acquired resistance, has prompted the research for therapeutic strategies targeting this pathway [37,38,39]. Dysregulation of Wnt/β-catenin pathway has been recognized to be associated with the development and progression of several types of cancers including breast cancer, ovarian cancer, and colorectal cancer [36,40,41]. In breast cancer, co-expression of Wnt ligands, including Wnt3, Wnt4, Wnt5a, and Wnt7a, leads to the activation of Wnt pathway [42], while in colorectal cancer, mutation or loss function of the *APC* gene or protein is more likely to be the reason for Wnt pathway activation [43]. Data from our current study indicated that Wnt3A, FZD6, and CTNNB1 were up-regulated, while GSK-3β was down-regulated in MKN45/R and NCI N87/R cells when compared to their parental cells, suggesting the acquirement of trastuzumab resistance through activation of canonical Wnt/β-catenin pathway in gastric cancer. Furthermore, specific modulation of Wnt/β-catenin pathway by *CTNNB1* knockdown reversed trastuzumab resistance, decreased cell proliferation, and increased apoptosis of trastuzumab-resistant gastric cancer cells, indicating the pivotal role of Wnt/β-catenin pathway in trastuzumab resistance of gastric cancer cells.

EMT has been widely recognized as a crucial process associated with cancer progression and drug resistance, during which cancer cells go through phenotypic alterations and acquire a higher potential for metastasis [44,45,46]. Since changes in cellular behavior are linked to EMT, we further investigated cell morphology and the expression of key EMT markers, showing an up-regulation of two mesenchymal markers (N-cadherin and vimentin) and a down-regulation of an epithelial marker (E-cadherin) in MKN45/R and NCI N87/R cells. Two transcription factors, snail1 and twist, which have been identified as master regulators of EMT, also showed a remarkable upregulation in the resistant cells. These data indicate that prolonged treatment of trastuzumab induces the EMT process, which is involved in trastuzumab resistance of gastric cancer cells.

Mounting evidence suggests that Wnt/β-catenin pathway plays an important role in EMT regulation of various cancers. In breast cancer, Wnt3 over-expression activates Wnt/β-catenin pathway which leads to trans-activation of EGFR and promotes EMT in trastuzumab-resistant cells [42]. In ovarian cancer, modulation of a single upstream gate-keeper of Wnt signaling, secreted frizzled related protein 4 (SFRP4), which functions as a tumor suppressor, can activate Wnt signaling and promote EMT [47]. Our results suggest that over-expression of Wnt3A and FZD6 activates the Wnt/β-catenin pathway in trastuzumab resistant gastric cancer cells, which is accompanied with EMT. Moreover, ICG-001, a specific inhibitor of β-catenin signaling, dramatically reduced phosphor β-catenin and reversed EMT, demonstrating that inhibition of Wnt/β-catenin signaling attenuates EMT in MKN45/R and NCI N87/R cells, suggesting that Wnt/β-catenin signaling is one of the major pathways involved in EMT that plays an integral role in trastuzumab-acquired resistance. Our current study concludes that Wnt/β-catenin pathway could be considered as a target for trastuzumab-resistant gastric cancer, and Wnt/β-catenin pathway inhibitors coupled with trastuzumab may help conquer resistance and improve the effects of trastuzumab in gastric cancer.

Moreover, pathway enrichment analysis in our study also uncovered some other pathways relevant to trastuzumab resistance which include insulin receptor, interleukin-17, Notch, Toll-like receptor cascades, Mitogen-activated protein kinase (MAPK) cascades, TGF-β, angiogenesis cascades, and focal adhesion pathways. Nevertheless, the underlying mechanisms of these pathways still require further investigation.

## 4. Materials and Methods

### 4.1. Cell Culture and Reagents

Human gastric cancer cell lines BGC823, MKN28, MGC803, SGC7901, and MKN45 were obtained from the State Key Laboratory of Proteomics (Beijing, China). The NCI N87 cell line was a generous gift from Academy of Military Medical Sciences (Beijing, China). All cells were maintained in Dulbecco’s Modified Eagle’s Medium (DMEM) (Gibco, New York, NY, USA) supplemented with 10% fetal bovine serum (FBS) (Gibco, USA) and 1% penicillin-streptomycin (Gibco) at 37 °C with 5% CO_2_.

### 4.2. Development of Trastuzumab-Resistant MKN45/R and NCI N87/R Gastric Cancer Cell Lines

Cells were cultured in DMEM medium containing 10 μg/mL trastuzumab (Roche, Basel, Switzerland) for four days. Cells that survived were cultured continuously under increasing concentration of trastuzumab (10, 20, 40, and 80 μg/mL) in the following five months. Cell proliferation was evaluated by CCK-8 (Dojindo, Kumamoto, Japan) assay at regular intervals. MKN45 and NCI N87 cells which grew stably in trastuzumab-containing medium (80 μg/mL) were sub-cultured successfully and therefore named MKN45/R and NCI N87/R, respectively.

### 4.3. Establishment of Stable Trastuzumab Resistant Cell Lines with β-Catenin Knockdown

β-catenin lentiviral siRNA plasmid (pGLV5-CTNNB1) and scrambled siRNA plasmid (pGLV5-NC) were acquired from Vipotion Biotechnology (Guangzhou, China). MKN45/R and NCI N87/R cell lines with β-catenin knockdown were established by infection of high titer lentiviral particles from GenePharma (Shanghai, China). Briefly, 293T cells were cultured in 6 cm plates with antibiotic-free media. When the confluence reached about 70%, cells were co-transfected with 2 μg packaging plasmid and 10 μg of siRNA plasmid mixed with lipofectamine 2000 (Invitrogen, Carlsbad, CA, USA) according to the manufacturer’s protocol. After incubation for 48 h, supernatants containing virus particles were collected by centrifugation. Target cells were transduced with virus particles by plating 1 × 10^5^ cells/well in a 6-well plate. Knockdown efficiency was assessed by RT-PCR and Western blot. Stable cell lines with β-catenin knockdown were used in the subsequent study.

### 4.4. Resistance Index Assays

Cells were incubated in 96-well plates at a density of 5 × 10^3^ cells/well and cultured for 24 h. Then cells were exposed to trastuzumab under different concentrations. Absorbance (*A*) was detected using CCK-8 in a microplate reader at a wavelength of 450 nm at regular intervals. Cell viability was measured according to the following formula: (*A*_(drug–supplemented)_ − *A*_(blank)_)/(*A*_(normal)_ − *A*_(blank)_) × 100%. Cell inhibition rate (%) =100% − Cell viability. The half maximal inhibitory concentration (IC_50_) was calculated using GraphPad Prism 5.0 software. The resistance index was calculated according to the following formula: Resistance index = IC_50_ (resistant cells)/IC_50_ (parental cells).

### 4.5. Wound Healing Assays

Cells were cultured in 6-well plates. After 24 h of incubation wounds were created by scraping the cells with a 200 μL pipette tip. Cells were then washed twice with phosphate-buffered saline (PBS) to clear away debris at the edge of the scratch and subsequently incubated in DMEM with 10% FBS. Microscopic images of the wounds at different time points were captured by the Leica DMi8 microscope system (Leica Microsystems, Wetzlar, Germany). The scratch assay was performed in triplicate.

### 4.6. Colony Formation Assays

About 5 × 10^3^ cells/well were seeded in 6-well plates and incubated in DMEM containing 3% FBS for 14 days, Afterwards, cells were washed three times with PBS, fixed with cold methanol for 15 min, and stained with 0.1% crystal violet. Colonies were photographed and counted. The assay was repeated three times.

### 4.7. Immunofluorescence Staining

Immunofluorescence assays were done as previously described [30]. Briefly, cells were washed 3 times with cold PBS, and fixed in 10% formaldehyde for 15 min, after which cells were permeabilized in 0.5% Triton X-100 for 30 min, and blocked in 2.5% bovine serum albumin (BSA) for 0.5 h. Subsequently, cells were incubated with primary antibody at 4 °C, then with secondary antibody conjugated with Alexa Fluor^®^ 594 (Santa Cruz Biotechnology, #sc-516642, Santa Cruz, CA, USA) or 488 (Santa Cruz Biotechnology, #sc-3895, USA) for 30 min at 37 °C. Finally, coverslips were counterstained with DAPI (Beyotime, Haimen, China) to visualize nuclei. Images were captured under Leica DMi8 fluorescence microscope.

### 4.8. Viability and Apoptosis Assays

Cells were seeded at 5 × 10^3^ cells/well into 96-well plates and cultured for 24 h. After treatment with a range of indicated concentrations of ICG-001(Medchem express, Princeton, NJ, USA) for 72 h, cell viability was measured using CCK-8 kit. Absorbance was detected at 450 nm with a microplate reader (Bio-Rad, Hercules, CA, USA). Apoptosis of cells was detected by dual staining with Annexin V-FITC/PI (CWBIO, #CW2574, Beijing, China) according to the manufacturer’s protocol. Briefly, cells were cultured for 24 h, and then treated with trastuzumab and harvested 96 h later. Cells were resuspended in binding buffer and stained with Annexin V-FITC/PI. After staining, cells were analyzed with the fluorescent activating cell sorting (FACS) Calibur system. All data were represented based on three independent experiments.

### 4.9. Reverse Transcription Polymerase Chain Reaction (RT-PCR)

Total RNA was extracted using Trizol reagent (Invitrogen, USA). The absorbance of RNA in RNase-free water was evaluated at 230, 260 and 280 nm using a Nanodrop 2000C spectrophotometer (Thermo, Waltham, MA, USA). The A260/A280 and A260/A230 ratios were maintained within the range of 1.80–2.10 and >2.0 respectively to ensure mRNA purity. One microgram (1 μg) of total RNA was treated with DNase I and the complementary DNA was amplified in vitro using SuperScript First-Strand cDNA Synthesis Kit (Invitrogen, USA). RT-PCR was subsequently performed using SYBR-Green Master Mix (Applied Biosystems, Foster City, CA, USA). The expression levels of target mRNAs were normalized to that of the control gene (β-actin). The sequences of primers used in this study are as follows: siRNA β-catenin forward: 5′-CCAGGAUGAUCCUAGC UAUTT-3′, reverse: 5′-AUAGCUAGGAUCAUCCUGGTT-3′; scrambled siRNA forward: 5′-GGAAGAUAAUCUUUUCUAATT-3′, reverse: 5′-UUAGAAAAGAUUAUCUUCCTT-3′; β-actin forward: 5′-GGACTTCGAGCAAGAGATGG-3′, reverse: 3′-GACATGCGGTTGT GTCACGA-5′.

### 4.10. Western Blotting

Cells were collected and lysed with radio-immunoprecipitation assay (RIPA) lysis buffer (50 mM Tris, 150 mM NaCl, 1% Triton X-100, 1% sodium deoxycholate, 0.1%sodium dodecyl sulfate (SDS), Beyotime) containing 1% protease inhibitor. Lysates were centrifuged at 12,000× *g* for 10 min at 4 °C, and the supernatant was collected for further use. Equal amounts (20–30 μg) of proteins were denatured in loading buffer and loaded on a gel after which SDS-PAGE was performed. Resolved bands were transferred to nitrocellulose membranes which were incubated with anti-HER-2 (Abcam, #16901, Cambridge, UK), anti-E-cadherin (Cell Signaling, #3195, Danvers, MA, USA), anti-N-cadherin (Cell Signaling, #13116, USA), anti-vimentin (Cell Signaling, #5741, USA), anti-snail1 (Cell Signaling, #3879, USA), anti-twist (Cell Signaling, #46702, USA), anti-PARP (Cell Signaling, #9532, USA), anti-β-catenin (Cell Signaling, #8480, USA), anti-*phosphor* β-catenin(Ser675) (Cell Signaling, #4176, USA), anti-Wnt3A (Abcam, #19925, UK), anti-FZD6 (Abcam, #98933, UK), anti-GSK-3β (Cell Signaling, #9315, USA), and β-actin (Cell Signaling, #4970, USA) respectively at 4 °C overnight followed by incubation with horseradish peroxidase (HRP)-labeled goat anti-rabbit or anti-mouse secondary antibodies (ZSGB-BIO, Beijing, China). Finally, the bands were visualized using enhanced chemi-luminescence reagent (CWBIO, China). The grey values of these target bands were measured using Image J software and histograms were plotted using GraphPad Prism software 5.0.

### 4.11. Cell invasion Assays

Following the established protocols [30], cell invasion assays were completed using a 24-well Transwell chamber containing polycarbonate filters with 8 μm pores which were coated with 60 μL 1:6 diluted Matrigel (BD Biosciences, San Jose, CA, USA). DMEM supplemented with 10% FBS was added to the lower chamber in the presence or absence of ICG-001 or 0.1% DMSO (as control), 3 × 10^4^ (NCI N87 and NCI N87/R) or 2 × 10^4^ (MKN45 and MKN45/R) cells/well in 200 μL of serum-free DMEM were added to the cell culture inserts. Cells were cultured for 22 h (for MKN45 and MKN45/R cells) or 28 h (for NCI N87 and NCI N87/R cells) respectively. The invaded cells passing through the filter were fixed with 10% formaldehyde, stained with 0.1% crystal violet and counted in six high power fields randomly selected under a light microscope.

### 4.12. Protein Extraction and Peptide Separation

Protein extraction and peptide separation were accomplished according to standard protocols as previously described [48,49]. Briefly, cells were lysed with RIPA buffer containing 1% protease inhibitors for 20 min at 4 °C. Protein concentration was determined by Bradford protein assay kit (Beyotime, China). Proteins extracted from each sample (100 μg) was digested with trypsin according to the filter-aided sample preparation (FASP) method [50]. Tryptic peptides were separated using C18 column in a pipette tip and eluted with increasing acetonitrile (6%, 9%, 12%, 15%, 18%, 21%, 25%, 30% and 35%). The nine separations were combined to six fractions, and dried in a vacuum concentrator.

### 4.13. Liquid Chromatography-Mass Spectrometry/Mass Spectrometry (LC-MS/MS) Analysis

Peptides were dissolved in solvent A (0.1% formic acid) and analyzed with FUSION mass spectrometer (Thermo) equipped with an Easy-nLC 1000 nanoflow high-performance liquid chromatography (HPLC) system (Thermo). Peptides were then separated on a reversed-phase C18 column (pre-column: 3 μm, 120 Å, 2 cm × 100 μm; analytical column: 1.9 μm, 120 Å, 12 cm × 150 μm) with an increasing gradient of 7–35% mobile phase B (0.1% formic acid in acetonitrile) at a flow rate of 600 nL/min for 75 min. A precursor scan was executed by scanning from *m*/*z* 300 to 1400 with a resolution of 120,000. Ions in each scan under top-speed mode were automatically isolated in Quadrupole with a 1.6 *m*/*z* window and fragmented by higher energy collision-induced dissociation with normalized collision energy of 35%. Dynamic exclusion was fixed for 18 s.

### 4.14. Protein Identification and Quantification

Raw files were searched against human Refseq protein database by Mascot 2.3 (Matrix Science Inc., Boston, MA, USA) implemented on Proteome Discoverer 1.4 (Thermo Scientific). The mass tolerances were 20 ppm for precursor and 0.5 Da for product ions. Two missed cleavages were allowed. The search engine set cysteine carbamidomethylation as a fixed modification, N-terminal acetylation, and oxidation of methionine as variable modifications. The data were accepted at a false discovery rate (FDR) of 1% at peptide level. Proteins quantification were done as our previous protocol [30,48,49]. Briefly, proteins were quantified using iBAQ approach, FOT was used to evaluate protein abundance, which was defined as a protein’s iBAQ divided by the total iBAQ of all proteins in one sample, and FOT was multiplied by 10^5^ for the ease of presentation.

### 4.15. Proteome Data Filtering and Analysis

Proteome data filtering was executed based on the following criteria. All keratins were excluded from our data and the rest of proteins detected in at least three of six experiments were used further for bioinformatics. Proteins were regarded as significantly changed in abundance if there was a more than twofold change between paired samples with a *p* value <0.05 using paired two-tailed student test. GO enrichment analysis was accomplished by WebGestalt. The significance level was set to *p* < 0.05 and all identified proteins as reference. Network analysis was done by the STRING, the interaction score was set to high confidence (scores > 0.7) while the networks were displayed by Cytoscape (v3.6.0).

## Supplementary Materials

Supplementary materials can be found at http://www.mdpi.com/1422-0067/19/7/1981/s1.

## Abbreviations

EMT	Epithelial-mesenchymal transition
HER-2	Human epidermal growth factor receptor-2
EGFR	Epidermal growth factor receptor
PI_3_K	Phosphatidylinositide 3-kinase
IGFR	Insulin-like growth factor receptor
PTEN	Phosphatase and tensin homolog gene
FGFR3	Fibroblast growth factor receptor 3
CBP	cyclic AMP response element-binding protein
β2-AR	β2-adrenergic receptor
iBAQ	Intensity based absolute quantification
FOT	Fraction of total
GO	Gene ontology
PCA	Principal component analysis
SFRP4	Secreted frizzled related protein 4
CCK-8	Cell Count kit-8
IC_50_	Half maximal inhibitory concentration
BSA	Bovine serum albumin
RT–PCR	Reverse transcription polymerase chain reaction
LC-MS/MS	Liquid chromatography–mass spectrometry/mass spectrometry
FDR	False discovery rate
FASP	Filter-aided sample preparation
