# Supplementary material for: Label-Free Quantitative Proteomics Combined with Biological Validation Reveals Activation of Wnt/β-Catenin Pathway Contributing to Trastuzumab Resistance in Gastric Cancer
Source: Int J Mol Sci. 2018 Jul 6;19(7):1981. doi: 10.3390/ijms19071981 (PMC6073113; doi:10.3390/ijms19071981)
Supplement: Supplementary file 1 [file ijms-19-01981-s001.zip › ijms-19-01981-s001/Supplementary materials/Figure S6.pdf]

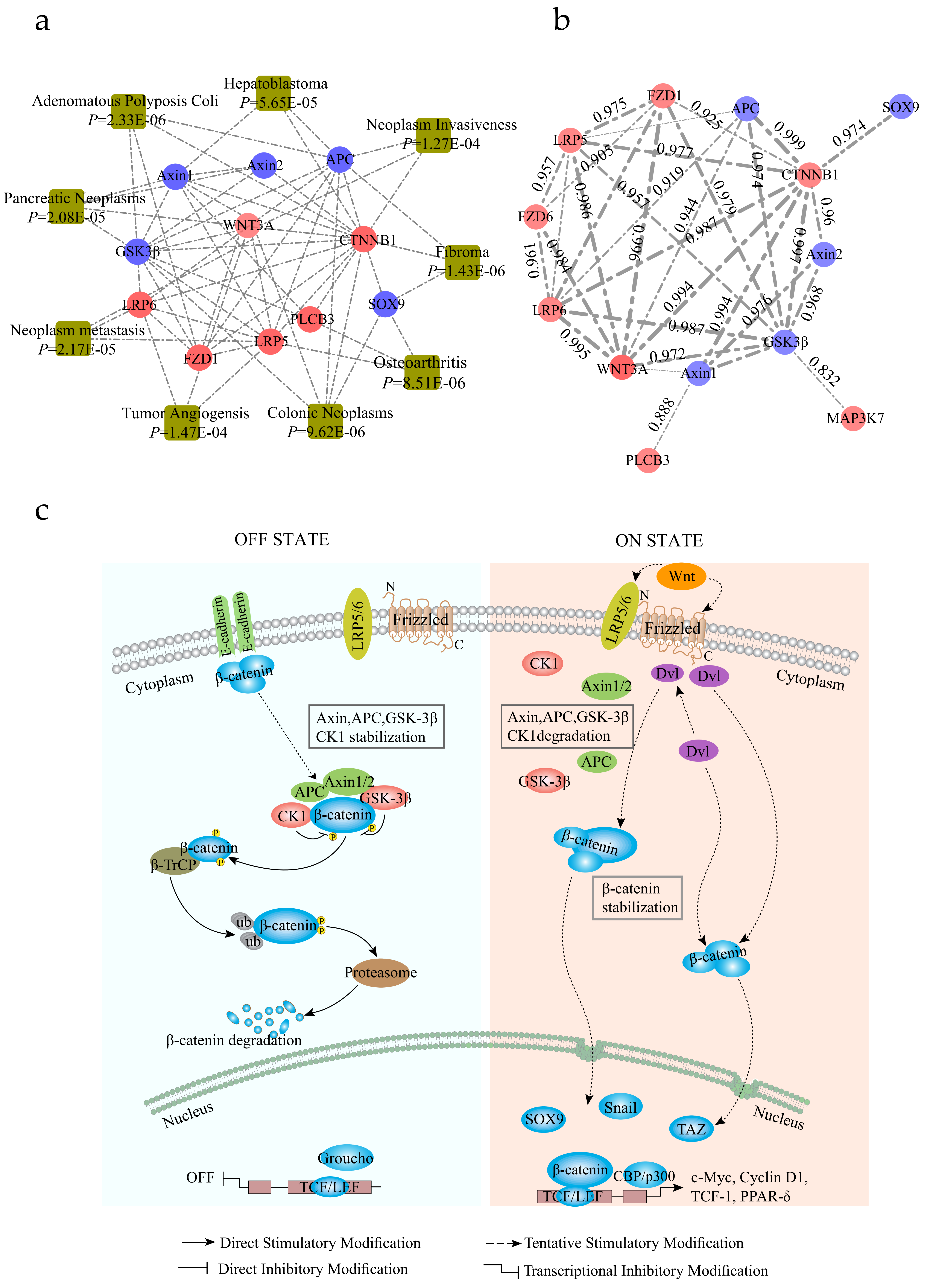

a
Adenomatous Polyposis Coli
P=2.33E-06
Hepatoblastoma
P=5.65E-05
Neoplasm Invasiveness
P=1.27E-04
Pancreatic Neoplasms
P=2.08E-05
Fibroma
P=1.43E-06
Neoplasm metastasis
P=2.17E-05
Osteoarthritis
P=8.51E-06
Tumor Angiogensis
P=1.47E-04
Colonic Neoplasms
P=9.62E-06
Axin1
Axin2
APC
WNT3A
CTNNB1
GSK3β
LRP6
PLCB3
SOX9
LRP5
FZD1
b
FZD1
APC
SOX9
LRP5
CTNNB1
FZD6
Axin2
LRP6
GSK3β
WNT3A
Axin1
MAP3K7
PLCB3
0.975
0.925
0.999
0.974
0.977
0.905
0.957
0.986
0.957
0.919
0.974
0.979
0.96
0.944
0.987
0.996
0.961
0.984
0.997
0.968
0.994
0.994
0.976
0.987
0.995
0.972
0.832
0.888
c
OFF STATE
ON STATE
E-cadherin
E-cadherin
LRP5/6
N
Frizzled
C
Cytoplasm
β-catenin
Axin,APC,GSK-3β
CK1 stabilization
APC
Axin1/2
GSK-3β
CK1
β-catenin
P
β-catenin
β-TrCP
ub
ub
β-catenin
Proteasome
β-catenin degradation
Nucleus
Groucho
OFF
TCF/LEF
Wnt
LRP5/6
N
Frizzled
C
CK1
Axin1/2
Dvl
Dvl
Cytoplasm
Axin,APC,GSK-3β
CK1degradation
APC
GSK-3β
Dvl
β-catenin
β-catenin
stabilization
β-catenin
SOX9
Snail
TAZ
Nucleus
β-catenin
CBP/p300
TCF/LEF
c-Myc, Cyclin D1,
TCF-1, PPAR-δ
Direct Stimulatory Modification
Tentative Stimulatory Modification
Direct Inhibitory Modification
Transcriptional Inhibitory Modification
