# Supplementary material for: Label-Free Quantitative Proteomics Combined with Biological Validation Reveals Activation of Wnt/β-Catenin Pathway Contributing to Trastuzumab Resistance in Gastric Cancer
Source: Int J Mol Sci. 2018 Jul 6;19(7):1981. doi: 10.3390/ijms19071981 (PMC6073113; doi:10.3390/ijms19071981)
Supplement: Supplementary file 1 [file ijms-19-01981-s001.zip › ijms-19-01981-s001/Supplementary materials/Supplementary materials1.doc]

**Supplementary Materials**

**Label-free quantitative proteomics combined with biological validation reveals activation of Wnt/β-catenin pathway contributing to trastuzumab resistance in gastric cancer**

**Wenhu Liu, Jiangbei Yuan, Zhenzhong Liu, Jianwu Zhang and Jinxia Chang**

**Figure S1**. Establishment of trastuzumab resistant gastric cancer lines.

(a) Detection of HER2 levels in gastric cancer cell lines NCI N87, MKN45, MKN28, BGC823, MGC803 and SGC7901 by western blot. (b, c) The *in vitro* inhibitor rate of MKN45, MKN45/R, NCI N87 and NCI N87/R cells were measured by CCK–8 assays. (d) Cells were incubated with different concentration trastuzumab (0, 60, 80 μg/mL) for 96 hours after which PARP and cleaved PARP were analyzed by western blot. (e) Detection of HER2 levels in NCI N87, NCI N87/R, MKN45 and MKN45/R cells by western blot. Data were presented as mean±SEM of three independent experiments.**P* <0.05, ***P* <0.01 *vs*. controls.

**Figure S2**. A workflow of differentially expressed proteins filtering.

**Figure S3**. Spearman’s correlation coefficients were calculated to assess the experimental reproducibility.

The lower-left half showed pairwise scatter plots of three biological repeats, with x and y axes representing log_2_ FOT intensity. The upper-right half showed pairwise spearman’s correlation coefficients for the same comparison.

**Figure S4.** PCA analysis was performed to distinguish MKN45 from MKN45/R cells.

**Figure S5**. The distribution of protein abundance ratios was displayed using histogram. The fold changes of MKN45/R cells/ MKN45 cells were shown in log_2_ scale on the x axis and the numbers of proteins on the y axis.

**Figure S6**. Bioinformatics analysis of differentially expressed proteins involved in Wnt/β-catenin pathway. (a) Diseases associated with differentially expressed proteins in Wnt/β-catenin pathway based on enrichment analysis by WebGestalt. Red and blue nodes represent up-regulated and down-regulated proteins respectively. (b) Network of differentially expressed proteins in Wnt/β-catenin pathway. Width of dotted lines and numbers represent the interaction confidence score. (c) Illustration of canonical Wnt/β-catenin signaling pathway showing key molecules utilized in the pathway. Left (Off state): In the absence of Wnt, a destruction complex, including Axin1/2, APC, GSK-3β and CK1, is located in the cytoplasm. β-catenin is dually phosphorylated by CK1 and GSK-3β after which it leaves the complex to be ubiquitinated by β-TrCP and is degraded by proteosome. Right (On state): In the presence of Wnt, signaling through the Frizzled receptor and LRP5/6 co-receptor complex induces the dual phosphorylation of LRP6 by CK1 and GSK-3β and allows for the translocation of a protein complex containing Axin1/2 from cytoplasm to plasma membrane. Dishevelled protein (Dvl) is recruited to the membrane and binds to Frizzled while Axin1/2 binds to phosphorylated LRP5/6. Frizzled/LRP5/6 complex formed at the membrane induces the stabilization of β-catenin by sequestration and/or degradation of Axin1/2. A portion of β-catenin translocates into the nucleus where it complexes with TCF/LEF family members to mediate transcriptional induction of target genes.

**Table S1**. List of all proteins identified and quantified in 3 replicates at 1% protein FDR with at least 2 unique and high quality peptides, all FOT showed here were multiplied by 10^5^.

**Table S2**. List of proteins were identified in at least 3 out of 6 experiments.

**Table S3**. List of all differentially expressed proteins and their values.

**Table S4**. List of differentially expressed proteins related to Wnt/β-canenin pathway and their values.
