# Supplementary figures and images for: Label-Free Quantitative Proteomics Combined with Biological Validation Reveals Activation of Wnt/β-Catenin Pathway Contributing to Trastuzumab Resistance in Gastric Cancer
Source: Int J Mol Sci. 2018 Jul 6;19(7):1981. doi: 10.3390/ijms19071981 (PMC6073113; doi:10.3390/ijms19071981)

**a**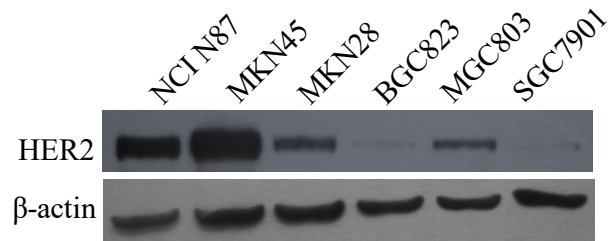**b**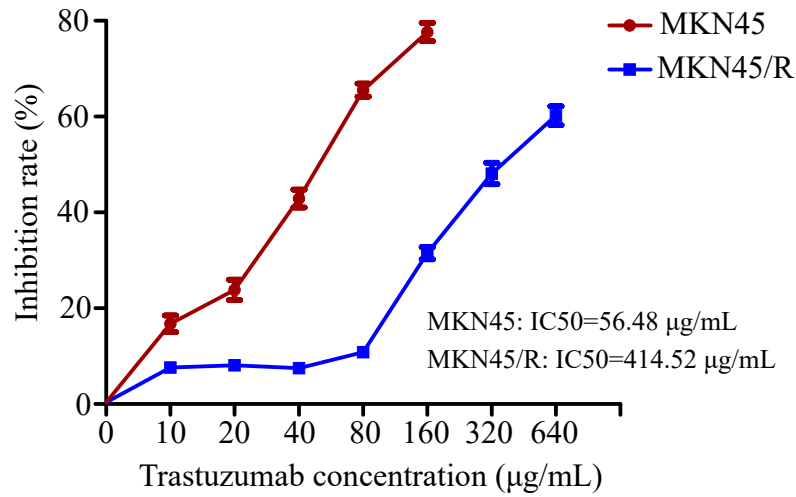**c**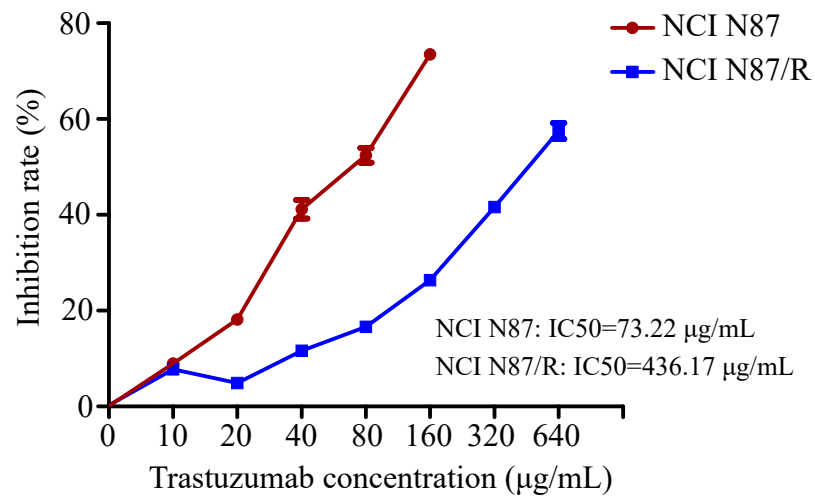**d**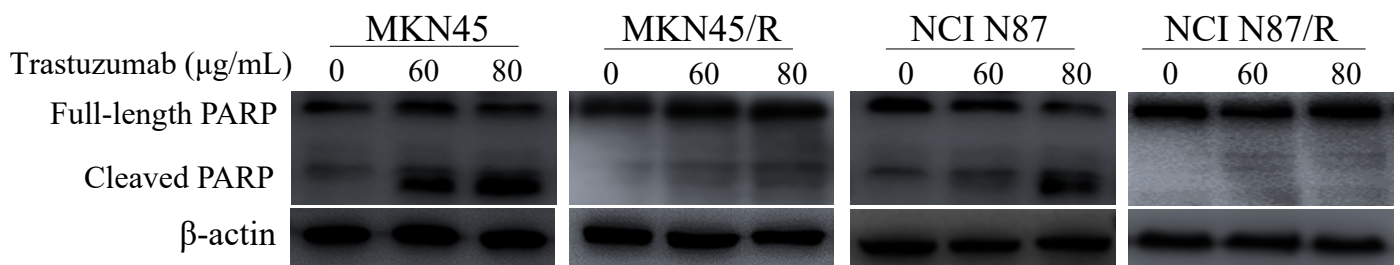**e**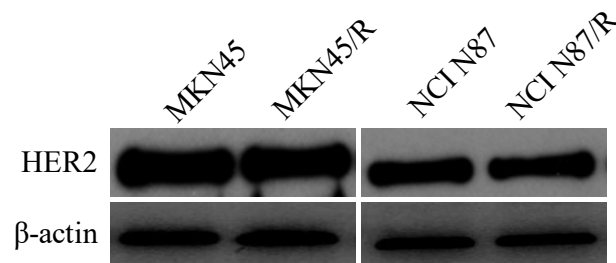

Supplement: Supplementary file 1 [file ijms-19-01981-s001.zip › ijms-19-01981-s001/Supplementary materials/Figure S1.pdf]

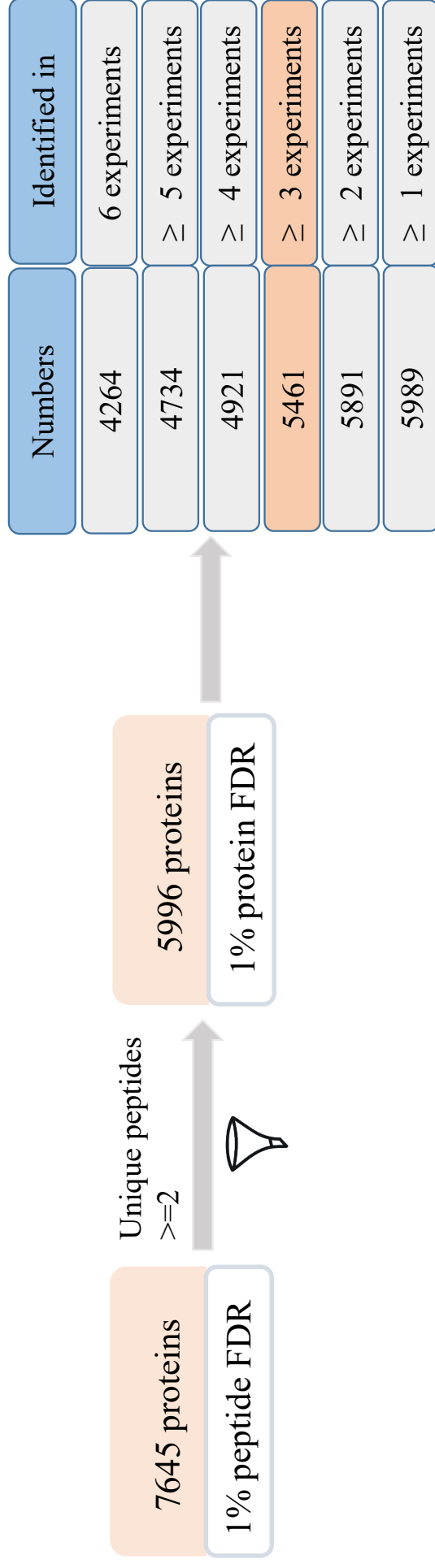

At least 3 out of 6 experiments

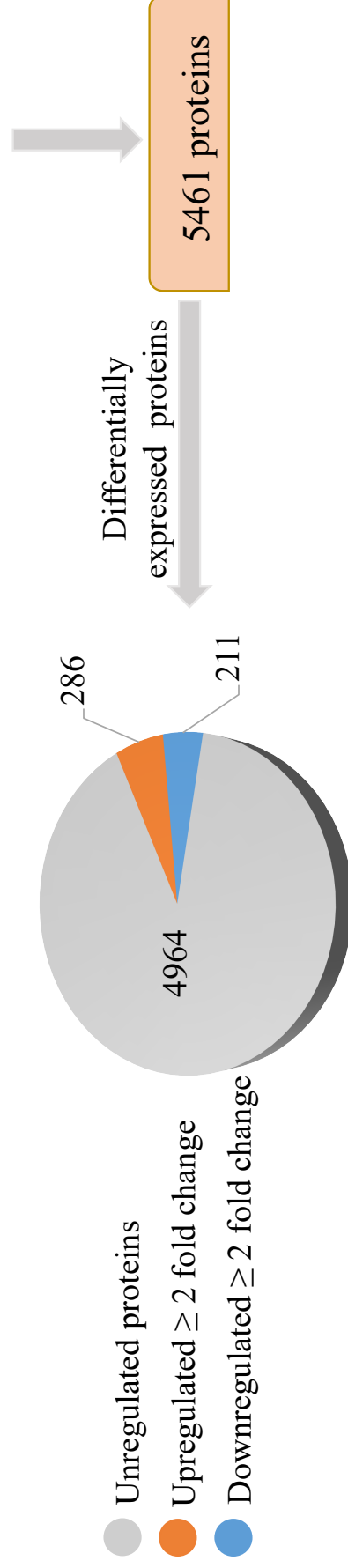

Supplement: Supplementary file 1 [file ijms-19-01981-s001.zip › ijms-19-01981-s001/Supplementary materials/Figure S2.pdf]

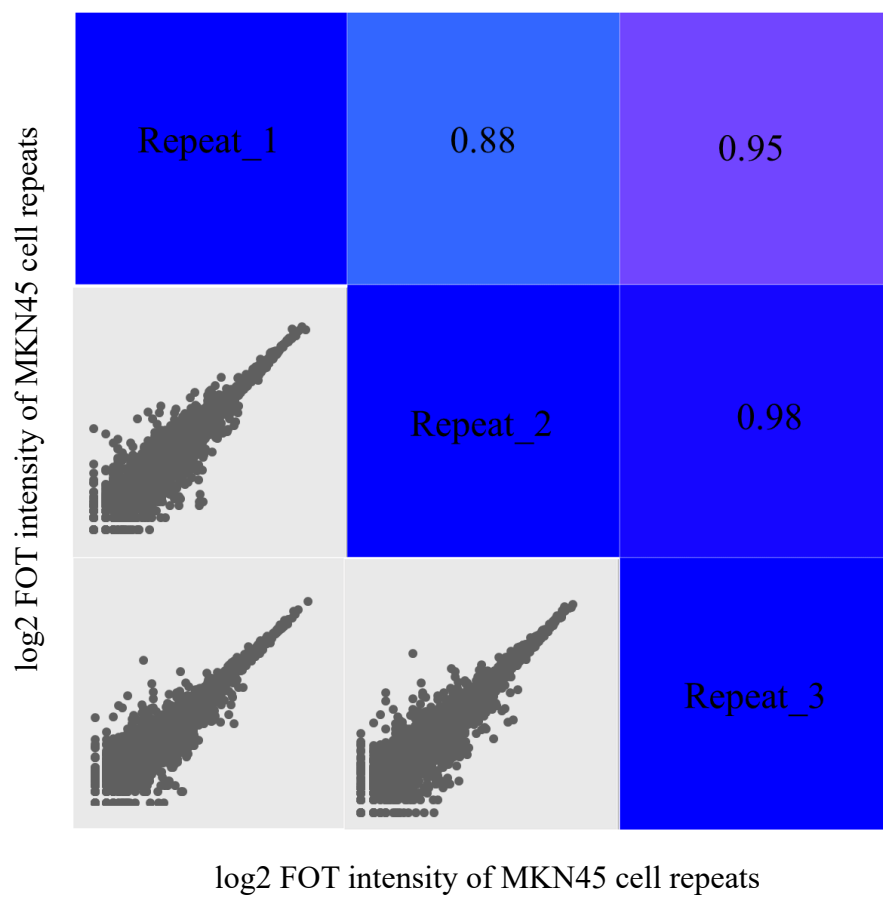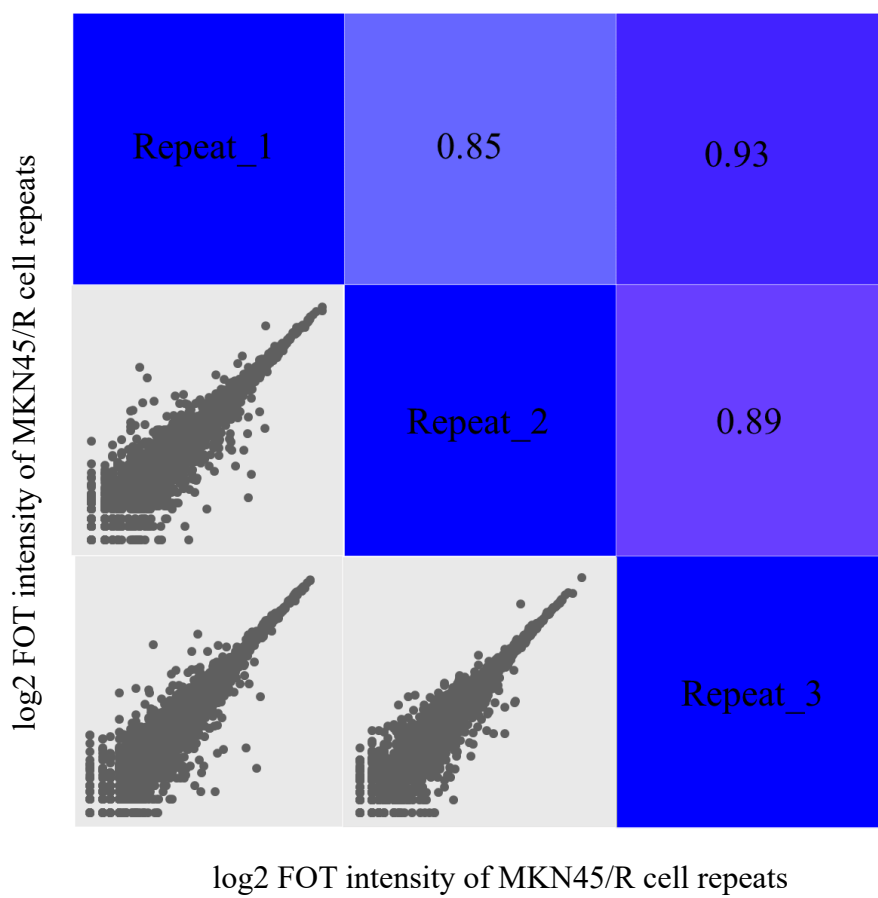

Supplement: Supplementary file 1 [file ijms-19-01981-s001.zip › ijms-19-01981-s001/Supplementary materials/Figure S3.pdf]

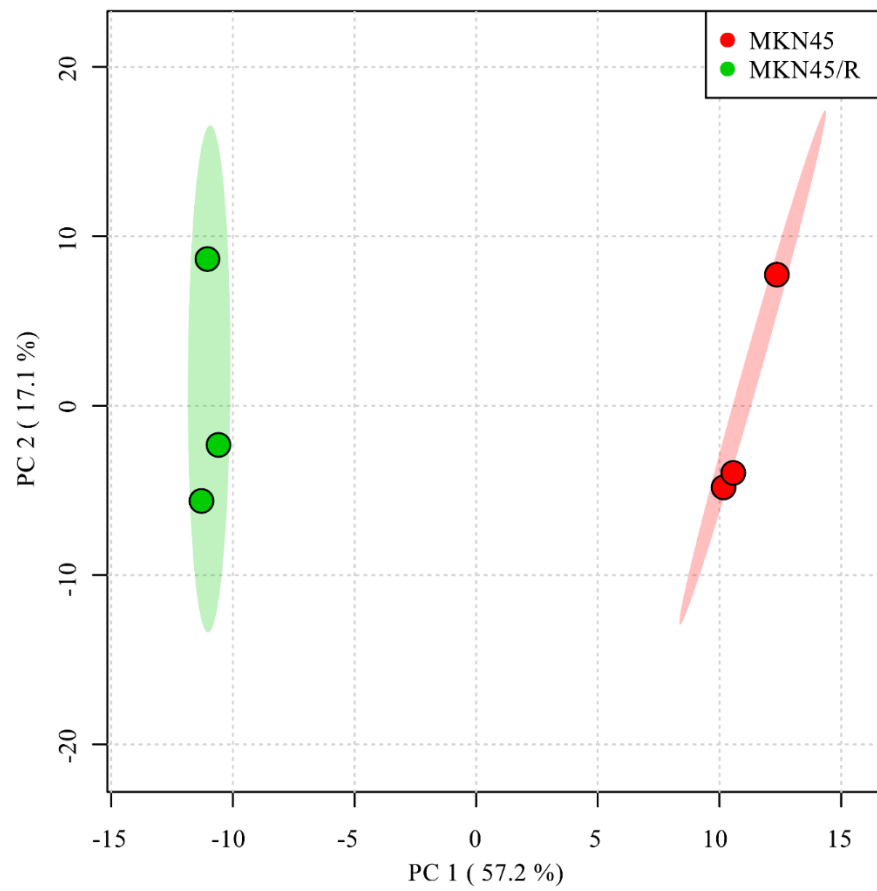

Supplement: Supplementary file 1 [file ijms-19-01981-s001.zip › ijms-19-01981-s001/Supplementary materials/Figure S4.pdf]

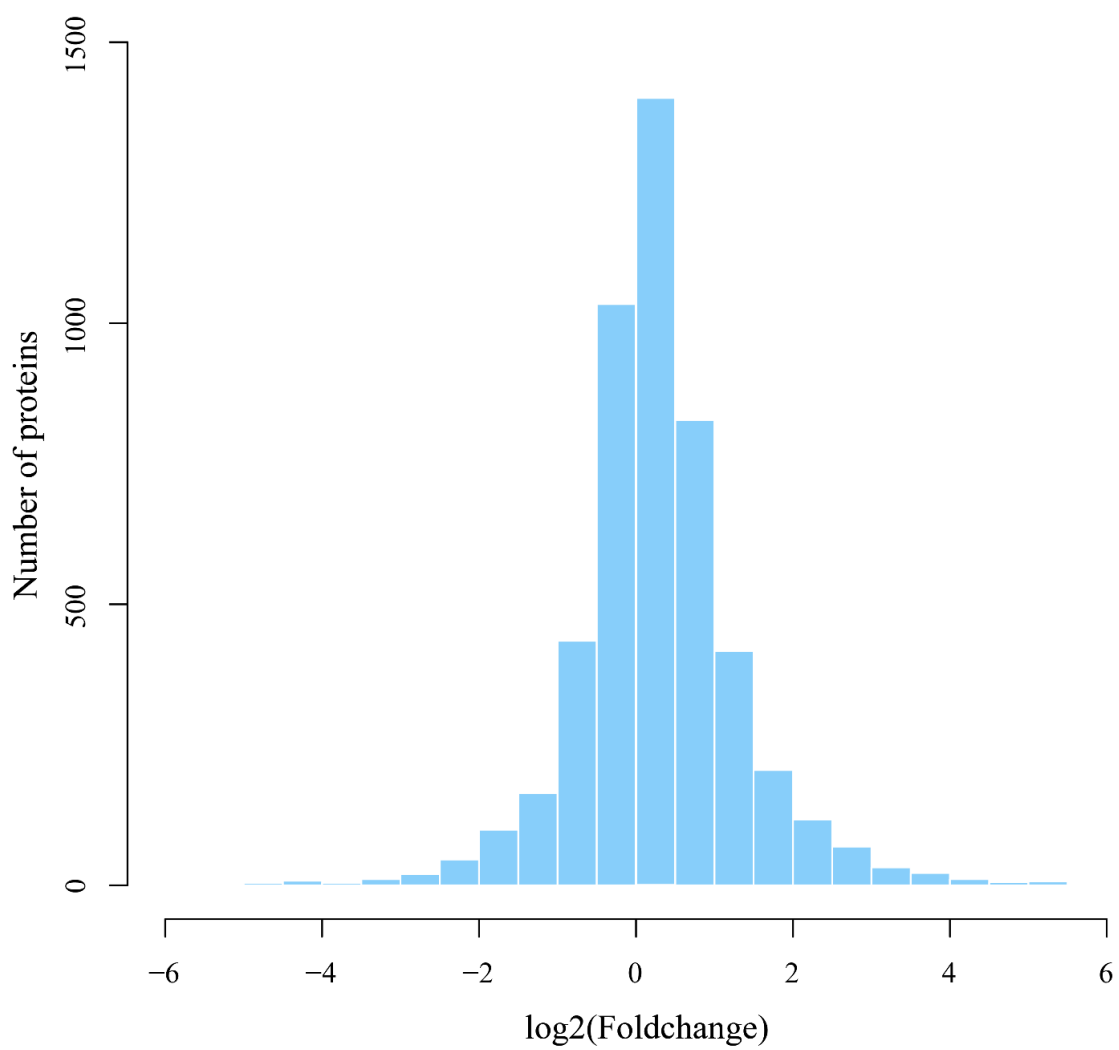

Supplement: Supplementary file 1 [file ijms-19-01981-s001.zip › ijms-19-01981-s001/Supplementary materials/Figure S5.pdf]
